# Postural Asymmetries and Assistive Devices Used by Adults With Cerebral Palsy in Lying, Sitting, and Standing

**DOI:** 10.3389/fneur.2021.758706

**Published:** 2021-12-06

**Authors:** Elisabet Rodby-Bousquet, Atli Agustsson

**Affiliations:** ^1^Centre for Clinical Research, Uppsala University—Region Västmanland, Västerås, Sweden; ^2^Department of Clinical Sciences Lund, Orthopaedics, Lund University, Lund, Sweden; ^3^Research Centre of Movement Science, School of Health Sciences, University of Iceland, Reykjavík, Iceland

**Keywords:** adults (MeSH), asymmetries, assistive devices, cerebral palsy, posture (MeSH), sitting position, standing position, supine position

## Abstract

**Purpose:** To describe the use of assistive devices and postural asymmetries in lying, sitting and standing positions in adults with cerebral palsy, and to analyze postural asymmetries and any associations with their ability to maintain or change position and time in these positions.

**Methods:** A cross-sectional study based on data from the Swedish Cerebral Palsy follow-up program of 1,547 adults aged 16–76 years, at Gross Motor Function Classification System (GMFCS) levels I (*n* = 330), II (*n* = 323), III (*n* = 235), IV (*n* = 298), and V (*n* = 361). Assistive devices such as wheelchairs, seating systems, adjustable beds, standing equipment and time in each position were reported. The Posture and Postural Ability Scale was used to identify asymmetries and rate the ability to maintain or change position. Binary logistic regression models were used to estimate odds ratios (OR) for postural asymmetries in supine, sitting and standing.

**Results:** Assistive devices were used by 63% in sitting (range 5–100% GMFCS levels I–V), 42% in lying (4–92% levels I-V), and 32% in standing (2–70% levels II–V). Wheelchairs were used as seating systems by 57%. Most adults had postural asymmetries in supine (75%; range 35–100% levels I–V), sitting (81%; 50–99% levels I–V) and standing (88%; 65–100% levels I–V). Men were more likely than women to have postural asymmetries, and the likelihood of postural asymmetries increased with age, GMFCS levels and inability to change position. Inability to maintain position increased the probability of postural asymmetries in all positions from OR 2.6 in standing to OR 8.2 in lying and OR 13.1 in sitting.

**Conclusions:** Almost twice as many adults used assistive devices in sitting than in lying or standing. Two thirds of the adults who used standing devices used it for <1 h per day, indicating that they might spend the remaining 23 out of 24 h per day either sitting or lying. Asymmetric postures were frequent across all ages and were highly associated with inability to change or maintain position.

## Introduction

Reduced postural ability is often a key problem in adults with cerebral palsy. Assistive devices can be used to accommodate a lack of stability and make it possible to maintain lying, sitting or standing positions (1, 2). Assistive devices for standing are used by 31% of children with cerebral palsy and for sitting by 42% (3) but little is known about the use of assistive devices to maintain position in adults with cerebral palsy.

Postural asymmetries are frequent and associated with a limited range of joint motion and an inability to change position (4, 5). Asymmetric postures sustained for long periods of time are associated with deformities, contractures and pain, which most commonly affect the spine and the lower extremities (5–8). Inability to move and having a sustained posture increases the likelihood for contractures, making each posture relative to the time spent in that position clinically important throughout a 24-h cycle (4, 5, 9). Supported standing programs for adults with neurological conditions have been suggested to improve range of motion and activity when used regularly at least 30 min, 5 times a week (10).

In 1994, Sweden introduced a follow-up program and registry for children with cerebral palsy called CPUP (11). In 2009, the program expanded and offered regular examinations also to adults with cerebral palsy. However, most adults currently enrolled have not previously been followed as children (4). The registry includes information on neurologic subtype, gross motor function, posture and postural ability, assistive devices used in sitting, standing and lying and time spent in these positions.

The purposes of this study were to describe the use of assistive devices and postural asymmetries in sitting, lying and standing positions in adults with cerebral palsy at all levels of motor function, and to analyze postural asymmetries and any association with their ability to maintain or change position and time in these positions.

## Methods

### Design and Inclusion

A cross-sectional study was performed based on data of all adults with cerebral palsy in Sweden, born between 1941 and 2002 who were examined within the follow-up program and reported into the registry between January 2016 and December 2018. Data from the last examination was used and no individual was excluded due to missing data. Inclusion criteria for cerebral palsy in this program were based on the Surveillance of Cerebral Palsy in Europe network with neurological symptoms of either spastic, ataxic, or dyskinetic cerebral palsy (12). Adults at all levels of the expanded and revised version of the Gross Motor Function Classification System level (GMFCS) I–V were included (13). GMFCS levels were classified by the local physical therapists at the examinations. Variables extracted from the follow-up program were type of assistive devices and time spent in sitting, standing and lying positions, postural asymmetries, ability to maintain and change position, and demographic variables such as sex, age and GMFCS level.

### Assistive Device and Time in Each Position

Assistive devices to maintain a body position were reported as “Yes or No” for lying and standing. Positioning equipment used for lying was reported as either adjustable bed or positioning cushions/rolls. Standing devices were specified as individually molded bilateral hip-knee-ankle-foot orthosis (HKAFO), standing frame/tilt board, or standing wheelchair. Equipment for mobility or transfers e.g., crutches, walkers or hoists were not included. For sitting, the options were either regular chair or assistive devices categorized as follows: custom molded seating systems; tilt-in-space wheelchairs (usually a recline manual or power wheelchair with high back rest); wheelchairs without tilt-in-space (usually a regular or active wheelchair); or adaptive seating (any modular seating system or adaptive seating prescribed as an assistive device to sit). A new variable was created based on all reported devices for sitting, with any assistive device to sit coded as “Yes” and a regular chair coded as “No.” Time spent standing was only reported for adults using standing devices into < 1, 1–2, 3–4, or >4 h, while time sitting and lying were reported for all individuals as <8, 8–12, or >12 h daily. Questions were asked by the therapists at the examinations and answered by the adult and/or proxy when needed.

### Postural Asymmetries and Ability to Change Position

Postural asymmetries and the ability to maintain and change position were assessed using the Posture and Postural Ability Scale (PPAS) (14). It shows high interrater reliability and validity when used with adults with cerebral palsy (14). The ability to maintain or change position was rated on a 7-point ordinal scale ranging from level 1 (unplaceable in an aligned position) to level 7 (able to move into and out of position independently). In this study, we refer to level 1 and 2 as “Cannot maintain position,” level 3 and 4 as “Maintains position,” level 5 and 6 as “Moves within position” and level 7 as “Changes position.” Postural asymmetries were rated in supine, sitting and standing positions, separately for the frontal (anterior-posterior) and the sagittal (medio-lateral) views. Symmetry of head, trunk, pelvis, leg, arm and foot position, and even weight distribution were scored as “1 point,” with a total score of 6 points for each position. Asymmetry or uneven weight distribution scored “0 points,” resulting in a total score ranging from 0 to 6 points. We refer to postural asymmetries as being “Severe” when the whole body is affected (0–1 point), as “Moderate” when 3 to 4 body segments are asymmetric (2–3 points), or as being “Mild” when 1 to 2 segments are asymmetric (4–5 points).

### Age and Sex

Age was grouped into six categories: 16–19, 20–24, 25–29, 30–39, 40–49, and 50–76 years, with a narrower age span in the younger age groups, when young adults transition from school and living with parents to higher education, employment and independent living (15). Sex was based on the legal gender, female or male.

### Ethical Approval

Ethical approval was granted by the Regional Ethical Review Board Lund, LU 443-99.

### Statistical Analyses

Spearman's rank correlation coefficients were used to estimate correlation coefficients among ordinal variables. Chi-square and Chi-square for trend were used to analyze differences between subgroups and increasing or decreasing trends in ordinal data (e.g., GMFCS levels). Binary logistic regression models were used to estimate odds ratios (OR) with 95% confidence intervals (CIs) for associations between postural asymmetries in lying, sitting or standing positions with their ability to maintain or change position in these positions, sex, age and GMFCS level. For the regression analyses, the primary outcome postural asymmetries was dichotomized into two groups, with 0 to 3 points graded as “Moderate and Severe asymmetry” and 4 to 6 points as “Mild or No asymmetry.” Interactions between adjusted variables were explored. The significance level was set to 0.05. The statistical analyses were performed using IBM SPSS Statistics for Windows, Version 26.0 (IBM Corporation, Armonk, NY).

## Results

In total, 1,547 adults with cerebral palsy were included, 854 men and 693 women, median age 25 (range 16–76 years). Most adults were classified as having bilateral spastic cerebral palsy (55.6%) and severe motor impairment, GMFCS V (23.3%) (Table 1).

**Table 1:** Description of all 1,547 participants with cerebral palsy.

		***N***	**%**
Sex	Male	854	55.2%
	Female	693	44.8%
Age (years)	16–19	304	19.7%
	20–24	450	29.1%
	25–29	288	18.6%
	30–39	251	16.2%
	40–49	142	9.2%
	50-76	112	7.2%
CP subtype	Spastic unilateral	335	22.0%
	Spastic bilateral	846	55.6%
	Ataxic	61	4.0%
	Dyskinetic	192	12.6%
	Mixed type	88	5.8%
	Missing	25	
GMFCS	GMFCS I	330	21.3%
	GMFCS II	323	20.9%
	GMFCS III	235	15.2%
	GMFCS IV	298	19.3%
	GMFCS V	361	23.3%

*GMFCS, Gross Motor Function Classification System*.

### Assistive Devices and Time in Each Position

**Figure 1:**
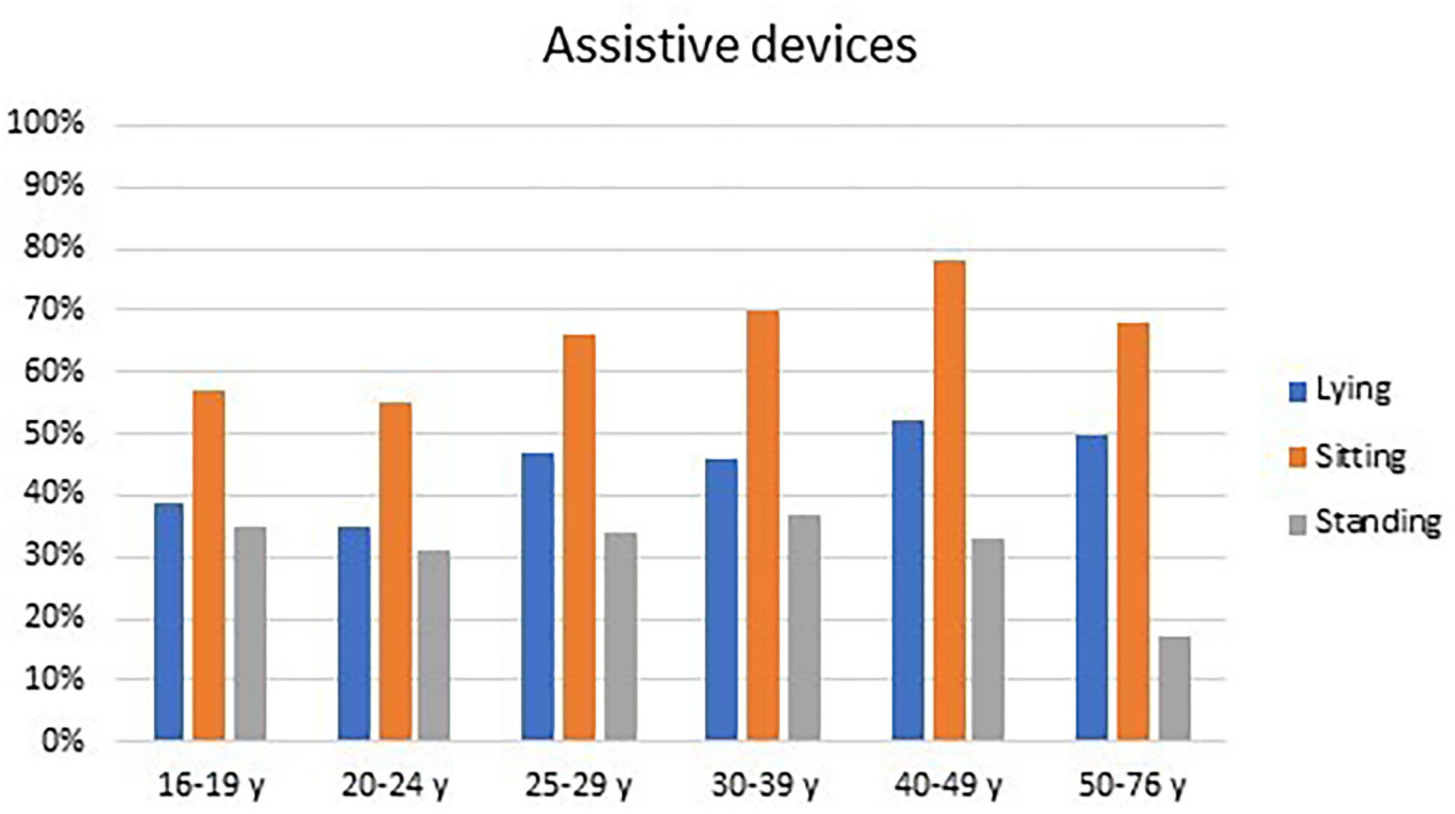
Assistive devices used in lying, sitting, and standing by adults at different age groups.

Assistive devices were used by 63% of all adults in sitting, by 42% in lying, and by 32% in standing. The use of assistive devices was similar for males and females but differed between age groups. The proportion of adults using assistive devices in lying and sitting was incrementally higher from 35 to 55% of the 20–24-year olds up to 52 and 78% of the 40–49-year olds (Figure 1). The use of standing devices ranged from 31 to 37% in the 16–49-year olds but dropped to 17% in the 50–76-year olds (Figure 1).

The use of assistive device increased with GMFCS level in sitting (*r*_*s*_= −0.78) and lying (*r*_*s*_= −0.67) (*p* < 0.001), from only 4–5% in those classified at GMFCS I, up to 92–100% of the adults at GMFCS V (Table 2). Positioning rolls or pillows were used by 31% of the adults and equally as many used adjustable beds (32%), with the vast majority being used by adults at GMFCS V. Wheelchairs were used as seating systems by 57%, by far the most common assistive device. Most individuals at GMFCS III and IV used regular wheelchairs, whereas the majority of those at GMFCS V used wheelchairs with tilt-in-space and almost half used a custom molded seating system. Standing devices were used by 32% of the adults and of those, 22% used either a standing frame or tilt-table, 12% used individually molded rigid bilateral HKAFO as their primary standing support, while 7% of adults used standing wheelchairs. The use of standing devices increased from 2% in adults at GMFCS II up to 70% of those at GMFCS V (*r*_*s*_= −0.62, *p* < 0.001) (Table 2).

**Table 2:** Assistive devices used in lying, sitting and standing by adults at GMFCS I to V.

	**GMFCS I**		**GMFCS II**		**GMCFS III**		**GMFCS IV**		**GMFCS V**		**Total**	
	**(*****n*** **=** **330)**		**(*****n*** **=** **323)**		**(*****n*** **=** **235)**		**(*****n*** **=** **298)**		**(*****n*** **=** **361)**		**(*****n*** **=** **1,547)**	
	***N***	**%**	***N***	**%**	***N***	**%**	***N***	**%**	***N***	**%**	***N***	**%**
Assistive devices in lying	15	4%	47	15%	79	34%	183	61%	332	92%	656	42%
Cushions, positioning rolls	11	3%	26	8%	43	18%	105	35%	290	80%	475	31%
Adjustable bed	4	1%	22	7%	47	20%	143	48%	282	78%	498	32%
Assistive devices in sitting	16	5%	99	31%	200	85%	298	100%	360	100%	973	63%
Moulded seating	0	0%	0	0%	6	3%	45	15%	170	47%	221	14%
Tilt in space wheelchair	0	0%	7	2%	30	13%	130	44%	277	77%	444	29%
Wheelchair no tilt	0	0%	48	15%	157	67%	190	64%	43	12%	438	28%
Adaptive seating	16	5%	70	22%	101	43%	88	30%	50	14%	322	21%
Assistive devices in standing	0	0%	7	2%	59	25%	181	61%	254	70%	501	32%
Standing frame/tilt board	0	0%	6	2%	44	19%	109	37%	181	50%	340	22%
HKAFO	0	0%	0	0%	10	4%	45	15%	135	37%	190	12%
Standing wheelchair	0	0%	3	1%	21	9%	58	20%	23	6%	105	7%

*GMFCS, Gross Motor Function Classification System; HKAFO, an individually molded rigid bilateral hip-knee-ankle-foot orthosis*.

Two thirds of the adults (67%) spent 8–12 h per day in bed, while 22% spent <8 h and 11% spent more than 12 h per day lying. Half of the adults (50%) spent 8–12 h per day sitting, while 27% sat <8 h and 22% sat more than 12 h per day. Almost two thirds (67%) used their standing support <1 h per day, while 28% were standing for 1–2 h daily and only 4% used it for more than 2 h daily. A slightly higher proportion of adults at GMFCS V (35%) spent at least 1 h standing daily compared with those classified at GMFCS III (28%) and IV (30%).

### Postural Asymmetries and Ability to Change Position

**Figure 2:**
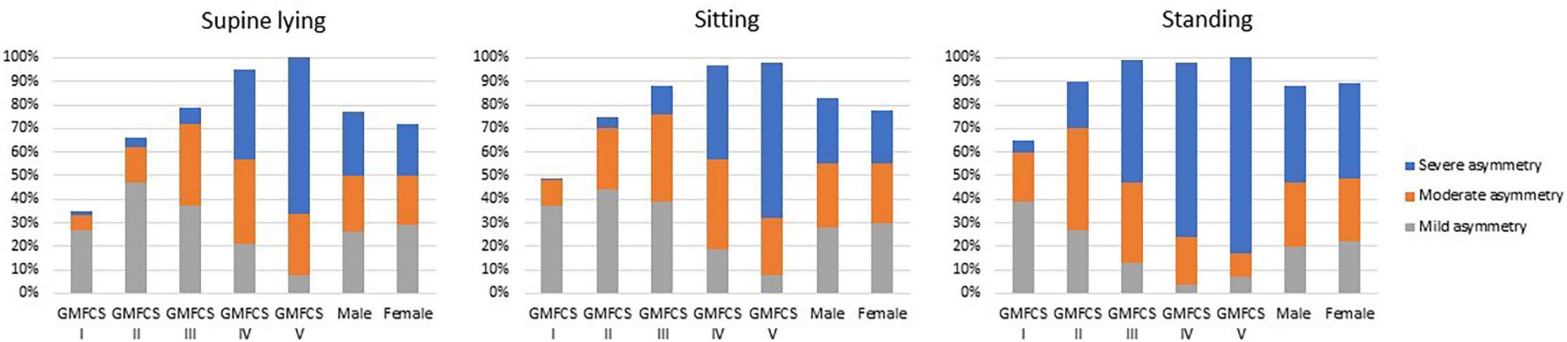
Postural asymmetries in supine lying, sitting, and standing positions of adults by GMFCS levels and sex.

Postural asymmetries were frequent in all positions and increased with GMFCS levels. In total, 75% of the adults had postural asymmetries in the frontal and/or sagittal view in lying, ranging from 35% of those at GFMCS I through 65% at level II, up to 95–100% of the adults at GMFCS levels IV and V. In sitting, 81% had postural asymmetries, ranging from 50% at GMFCS I up to 88% of the adults at level III and 98–99% at level IV and V, respectively. Almost nine out of 10 (88%) had postural asymmetries in standing, from 65% at GMFCS I and 90% at level II, to almost all adults at level III, IV and V (98–100%). A substantially higher proportion of the adults at GMFCS IV and V had severe asymmetries in all three positions compared to adults at GMFCS I and II (Figure 2).

As expected, postural ability decreased with lower levels of motor function and correlated significantly (*p* < 0.001) with the GMFCS in lying (*r*_*s*_= −0.82), sitting (*r*_*s*_= −0.87) and standing (*r*_*s*_= −0.86). All adults at GMFCS level I and II were able to change their position independently. Median values and 25 and 75th percentiles for postural asymmetry and postural ability are presented in Table 3. Individuals classified at GMFCS III showed the most variability between different positions, with most able to change position in lying but unable to maintain position without support in standing (Table 3).

**Table 3:** Median values with 25 and 75th percentiles for the Posture and Postural Ability Scale scores for posture and postural ability in supine, sitting and standing positions of adults at GMFCS I to V.

		**GMFCS I**		**GMFCS II**		**GMFCS III**		**GMFCS IV**		**GMFCS V**	
		**Median**	**Percentile**	**Median**	**Percentile**	**Median**	**Percentile**	**Median**	**Percentile**	**Median**	**Percentile**
Posture	Supine, frontal	6	(5–6)	5	(4–6)	5	(3–6)	2	(1–4)	1	(0–2)
	Supine, sagittal	6	(6–6)	6	(5–6)	5	(3–6)	3	(1–4)	2	(1–3)
	Sitting, frontal	6	(5–6)	5	(4–6)	4	(3–6)	2	(1–4)	1	(0–3)
	Sitting, sagittal	6	(5–6)	5	(4–6)	4	(3–5)	3	(2–4)	2	(0–3)
	Standing, frontal	5	(4–6)	4	(2–5)	2	(1–4)	1	(0–2)	0	(0–1)
	Standing. sagittal	6	(5–6)	4	(2–5)	2	(1–3)	1	(0–2)	0	(0–1)
Postural ability^*^	Supine lying	7	(7–7)	7	(7–7)	7	(7–7)	5	(4–7)	3	(3–4)
	Sitting	7	(7–7)	7	(7–7)	7	(6–7)	4	(2–5)	2	(2–2)
	Standing	7	(7–7)	7	(7–7)	6	(2–7)	2	(2–2)	2	(1–2)

*GMFCS, Gross Motor Function Classification System*.

* *Level 7, Able to move into and out of position; Level 6, Able to move out of position; Level 5, Able to transfer weight laterally and regain posture; Level 4, Able to initiate flexion/extension of trunk; Level 3, Able to maintain position when placed but cannot move; Level 2, Placeable in an aligned posture but needs support, Level 1, Unplaceable in an aligned posture (see section Postural Asymmetries and Ability to Change Position for more details)*.

Of the adults with severe asymmetries in lying, 95% of those at GMFCS V used lying support, while 76% of those at GMFCS IV and only 20% of those at GMFCS III did the same. Also, quite a high proportion of adults without asymmetries used lying support: 75% of those at level V and 40% at level IV. All individuals at GMFCS IV and V with severe asymmetries in sitting used seating support. In standing, all adults with severe asymmetries used standing support except those at GMFCS V, where only half (51%) used standing support.

Men were more likely than women to have postural asymmetries in supine (OR 1.77, 95% CI 1.34–2.35) and sitting (OR 1.37, 95% CI 1.05–1.79), and the likelihood of postural asymmetries increased with age, GMFCS levels and inability to change position (Table 4). Inability to maintain position increased the likelihood of an asymmetric posture in both supine (OR 8.18, 95% CI 2.92–22.95), sitting (OR 13.1, 95% CI 6.26–27.41) and standing positions (OR 2.62, 95% CI 1.38–4.97) even when adjusted for age, sex, and GMFCS level (Table 4).

**Table 4:** Odds ratios (OR) with 95% confidence intervals (CI) and R square (*R*^2^) estimated for moderate to severe postural asymmetries in lying, sitting and standing positions.

**Postural asymmetries**	**Supine lying (*****R***^**2**^ **=** **0.52)**				**Sitting (*****R***^**2**^**=** **0.47)**				**Standing (*****R***^**2**^ **=** **0.37)**			
	**OR**	**95% CI**		***P*-value**	**OR**	**95% CI**		***P*-value**	**OR**	**95% CI**		***P*-value**
Female	*Ref*				*Ref*							
Male	1.77	1.34	2.35	<0.001	1.37	1.05	1.79	0.021	1.21	0.93	1.59	0.162
Age	1	0.99	1.01	0.743	1.01	1.00	1.03	0.014	1.01	1.00	1.03	0.043
GMFCS I	*Ref*				*Ref*				*Ref*			
GMFCS II	2.44	1.44	4.11	0.001	3.50	2.15	5.73	<0.001	3.21	2.25	4.58	<0.001
GMFCS III	5.64	3.34	9.51	<0.001	4.92	2.89	8.37	<0.001	5.45	3.35	8.88	<0.001
GMFCS IV	15.50	8.68	27.69	<0.001	9.13	4.69	17.77	<0.001	9.06	4.29	19.14	<0.001
GMFCS V	27.36	13.45	55.64	<0.001	5.47	2.33	12.80	<0.001	14.93	6.27	35.50	<0.001
Changes position	*Ref*				*Ref*				*Ref*			
Moves within position	1.73	1.08	2.77	0.023	1.53	0.91	2.57	0.106	1.23	0.49	3.08	0.667
Maintains position	3.36	1.97	5.74	<0.001	3.26	1.81	5.86	<0.001	1.35	0.59	3.12	0.481
Cannot maintain position	8.18	2.92	22.95	<0.001	13.10	6.26	27.41	<0.001	2.62	1.38	4.97	0.003

*Binary logistic regression models with all variables adjusted for all other variables in the model*.

*GMFCS, Gross Motor Function Classification System*.

## Discussion

This study describes the use of assistive devices in lying, sitting and standing positions in adults with cerebral palsy at all GMFCS levels, postural asymmetries and associations with their ability to maintain or change position and time in these positions.

### Assistive Device and Time in Each Position

The use of assistive devices in standing (32%) was similar to previous findings reported for children (31%) (3), while the use of assistive devices in sitting was substantially higher in adults (63%) compared with children (42%) (3). The use of wheelchairs as seating systems (57%) in adults can also be associated with an increased use of wheelchairs for mobility with age, as previously reported for children and adolescents with cerebral palsy (16, 17). Transfers between different assistive devices may also be more challenging and time-consuming in adults than in younger children and may lead to the use of wheelchairs as a seating solution and not only as mobility equipment in adults. In addition, lying support was used by 42% of adults. We found no comparable data of lying support for children.

The use of assistive devices was similar for men and women. If we look more closely at the adults at GMFCS IV and V, everyone used assistive devices to sit, and a vast majority also used lying support (61–92%), which indicates that a majority of adults with cerebral palsy have postural support most of the day and night. Proper use of adjustable beds and positioning equipment such as rolls and pillows should provide comfortable non-harmful postures in lying, increase the weight-bearing area to improve sleep and minimize pain and pressure. A significant improvement in sitting posture and postural control has previously been found in people with cerebral palsy using seating support, such as orthotics, seat inserts, external supports and modular seating systems (18–20). We found that all adults at GMFCS level IV and V who had an asymmetric sitting posture used an assistive device in sitting, which is encouraging. Almost half of those at GMFCS V used custom molded seating systems and two thirds used tilt-in-space wheelchairs. These are provided for free and the total annual fee for visits to therapists, general practitioners, assistive technology centers, primary health care or hospitals for adults in Sweden is limited to 113 euros.

We found that three in four adults spent at least 8 h of the 24 h in a day lying (76%) and 8 h or more in sitting (74%). Almost two thirds (67%) of the adults who used standing support used it <1 h per day, which is similar to the 64% previously reported for children in the UK (21). This finding is concerning, as it implies that adults who use standing devices spend 23 out of the 24 h per day either sitting or lying. In addition, most of the adults using standing support (GMFCS IV and V) were unable to change their position independently while lying or sitting. The time spent in each position is more important for those who cannot change position independently, as they are more likely to remain in the same position over long periods (5). The opportunity for a change in position was reported as an indication of a need for standing devices in children by almost 80% of the parents and clinicians in the UK (21).

### Postural Asymmetries and Ability to Change Position

Postural asymmetries were more frequent and involved more body segments in a standing position than in sitting or lying. This contrasts with previous findings in young adults with cerebral palsy where those at GMFCS level V were reported to have fewer asymmetries in standing compared with sitting and lying (4). This may be partly explained by the difference in sample size (102 vs. 1,547) and age span (19–23 vs. 16–76 years). Alternatively, it might indicate that the asymmetries seen in young adults might be more reducible when provided with appropriate support, while the asymmetries in older adults might be more associated with non-correctable fixed deformities. A recent study of children with cerebral palsy shows an increasing trend, with more asymmetries in older children and adolescents than in younger children (7). However, the asymmetries were already seen in children before the age of 3 years (7). This is a major concern, as asymmetric posture in early life is associated with the development of fixed deformities such as windswept hips and scoliosis (22). Also, contractures have a tendency to develop over time in individuals with cerebral palsy (23) and they increase the risk of fixed deformities (24).

Despite challenges with asymmetric standing postures, the use of assistive devices in a standing position was almost the same in adults as previously reported for children. Our results indicate that a reduced ability to change and maintain a position increases the likelihood of an asymmetric posture. As noted above, asymmetric postures in sitting and lying are associated with scoliosis and windswept deformity in both children and adults with cerebral palsy (5, 8, 25), which might be explained by the longer time spent in these two positions. Several adults without asymmetries used adjustable beds and positioning equipment in lying. Hopefully this is a sign of proactive rather than reactive treatment strategies.

Maintaining or changing a standing position is normally more challenging, compared with sitting and supine positions. This observation is in line with our findings where the median scores and 25 and 75th percentiles for postural ability in standing were lower for adults at GMFCS levels III to V compared with those in supine and in sitting. There was also a clear trend for lower postural ability for adults at GMFCS III, IV and V. Not being able to maintain or change position can lead to the development of contractures and deformities (5). Individuals with cerebral palsy at GMFCS levels IV and V, accounting for a total of 43% of our cohort, were unable to maintain or change position independently and thereby are at a high risk of developing contractures and deformities. We found postural asymmetries across all GMFCS levels, but they were more frequent in adults at GMFCS IV and V. Surprisingly, men were more likely than women to have postural asymmetries in both supine and sitting positions, but their use of assistive devices to stay in these positions were similar. Moreover, inability to maintain or change position was identified as an independent risk factor and significantly increased the likelihood for asymmetric postures in all three basic body positions even when adjusted for age, sex and GMFCS levels.

### Limitations

The cross-sectional design cannot establish any causal relationships but only identify associations between variables. Even though we had access to a relatively large cohort, including adults classified at all functional levels and with a wide age span, it does not represent the total population of adults with cerebral palsy. The age distribution of our cohort is skewed, with a preponderance of younger adults under 30 years of age. There is a slightly higher proportion of adults with spastic bilateral cerebral palsy and less with spastic unilateral cerebral palsy than reported by others (26–28). There is also a higher proportion of adults with more severe motor impairment classified at GMFCS V and less at GMFCS I compared with the distribution of GMFCS levels reported for children (27). We cannot say if this is a selection bias or an indication of the decline in gross motor function seen in adults with CP. In Sweden, assistive devices such as lying support and sitting and standing support, are usually provided as a loan by regional Assistive Technology Centers. Therefore, the results of this study are likely to reflect the use of assistive devices without regard to the socioeconomic situation of the individuals. Even though this study represents un unselected population of adults with CP reported into the registry regardless of their age, sex, motor function, communication and cognitive abilities or neurological impairment, the findings may not be representative for other countries with different healthcare systems. All data is retrieved from a National Registry and as such, covers the whole country. Even though all data is reported into the database according to established guidelines and manuals, there might be some errors due to classification or reporting errors. Time spent in different positions was self-reported and/or reported by proxy. This could potentially affect the results as the agreement between these two has not been evaluated. Even so, the use of GMFCS and PPAS has previously shown a high reliability and validity for adults with cerebral palsy (14, 29).

## Conclusions

In this study we found that two out of three adults with cerebral palsy used assistive devices in sitting, which was almost twice as many as those who used assistive devices in lying and standing. Most adults used their wheelchairs as seating systems. Two thirds of the adults who used standing devices used it for <1 h per day, indicating that they might spend the remaining 23 out of 24 h per day either sitting or lying. Standing is normally more challenging and postural asymmetries were more frequent and involved more body segments in standing than in sitting or lying. An unexpected finding was that men were more likely than women to have postural asymmetries. We found postural asymmetries across all GMFCS levels, but they were more frequent in adults at GMFCS IV and V and they were highly associated with inability to change or maintain position. Therefore, it should be a priority to facilitate more frequent changes in position for those who cannot change position on their own. However, it is encouraging that several adults without asymmetries used adjustable beds and positioning equipment in lying. Hopefully this is a sign of proactive rather than reactive treatment strategies and efforts to prevent the development of postural asymmetries.

## Data Availability Statement

The datasets presented in this article are not readily available because the data analyzed in this study were obtained from the Cerebral Palsy Follow-Up Program (CPUP) registry, the following licenses/restrictions apply: requests to access the datasets are subject to ethical approval and must first be granted by KVB Region Skåne. Requests to access the datasets should be directed to https://vardgivare.skane.se/kompetens-utveckling/forskning-inom-region-skane/utlamnande-av-patientdata-samradkvb/.

## Ethics Statement

The studies involving human participants were reviewed and approved by Regional Ethical Review Board Lund. Written informed consent from the participants' legal guardian/next of kin was not required to participate in this study in accordance with the national legislation and the institutional requirements.

## Author Contributions

ER-B and AA designed the study, analyzed the data, drafted the manuscript, and approved the final draft. Both authors contributed to the article and approved the submitted version.

## Funding

This study was supported by grants from FORTE—the Swedish Research Council for Health, Working Life and Welfare, Grant No: 2018-01468.

## Conflict of Interest

The authors declare that the research was conducted in the absence of any commercial or financial relationships that could be construed as a potential conflict of interest.

## Publisher's Note

All claims expressed in this article are solely those of the authors and do not necessarily represent those of their affiliated organizations, or those of the publisher, the editors and the reviewers. Any product that may be evaluated in this article, or claim that may be made by its manufacturer, is not guaranteed or endorsed by the publisher.
